# Methods of estimating prevalence of multiple sclerosis in six European healthcare data sources: a contribution from the ConcePTION project

**DOI:** 10.1007/s10654-025-01243-8

**Published:** 2025-07-31

**Authors:** Marie Beslay, Anna-Belle Beau, Davide Messina, Justine Benevent, Elisa Ballardini, Laia Barrachina-Bonet, Clara Cavero-Carbonell, Alex Coldea, Laura García-Villodre, Anja Geldhof, Rosa Gini, Kerstin Hellwig, Sue Jordan, Maarit K. Leinonen, Sandra Lopez-Leon, Marco Manfrini, Visa Martikainen, Vera R. Mitter, Amanda J. Neville, Hedvig Nordeng, Aurora Puccini, Sandra Vukusic, Christine Damase-Michel, Yvonne Geissbühler, Joan K. Morris

**Affiliations:** 1 CERPOP-SPHERE Team, Toulouse University Hospital, Inserm UMR 1295, Toulouse University, Toulouse, France; 2 ARS Toscana, Florence, Italy; 3 Neonatal Intensive Care Unit, Department of Medical Sciences, Centre for Clinical and Epidemiological Research, Emilia Romagna Registry of Birth Defects, University Hospital of Ferrara, University of Ferrara, Ferrara, Italy; 4 Rare Disease Research Unit, Foundation for Promotion of Health and Biomedical Research of Valencian Region (FISABIO), Valencia, Spain; 5 Faculty of Medicine, Health and Life Sciences, Swansea University, Swansea, Wales; 6 Janssen Biologics B.V, Leiden, Netherlands; 7 Department of Neurology, St. Josef-Hospital, Ruhr-University, Bochum, Germany; 8 Department of Data and Analytics, Finnish Institute for Health and Welfare, Helsinki, Finland; 9 Novartis Pharmaceuticals Corporation, East Hanover, USA; 10 Rutgers Center for Pharmacoepidemiology and Treatment Science, Rutgers University, New Brunswick, NJ USA; 11 Department of Medical Sciences, Centre for Clinical and Epidemiological Research, Ferrara University, Ferrara, Italy; 12 School of Health Professionals, Midwifery Department, Bern University of Applied Sciences, Bern, Switzerland; 13 IMER Registry (Emilia Romagna Registry of Birth Defects), University Hospital of Ferrara, Ferrara, Italy; 14 Pharmacoepidemiology and Drug Safety Research Group, Department of Pharmacy, Faculty of Mathematics and Natural Sciences, University of Oslo, Oslo, Norway; 15 Emilia-Romagna Regional Center of Pharmacovigilance, Bologna, Italy; 16 Hospices Civils de Lyon, Hôpital Pierre Wertheimer, Université Claude Bernard Lyon-I, Research on Healthcare Performance, INSERM U1290, Bron, Lyon, France; 17 Evidence Generation, Novartis Pharma AG, Basel, Switzerland; 18 Population Health Research Institute, City St George’s , University of London, London, UK

**Keywords:** Prevalence calculation methods, Lookback, Multiple sclerosis, Administrative healthcare data sources, Women of childbearing age, Pregnant women

## Abstract

Multiple sclerosis (MS) is a chronic autoimmune condition primarily affecting women and often diagnosed during childbearing years. This study assessed the impact of the lookback period and calculation method on MS prevalence in three healthcare data sources including women of childbearing age (from Italy, Norway and Wales) and three data sources including pregnant women (from France, Finland and Spain). Women aged 15 to 49 years from 2005 to 2019 were included, data from pregnant women were collected around the pregnancy period. MS cases were identified based on at least one MS diagnosis or one dispensation for an MS-specific medication. All data sources provided inpatient diagnoses and medication data; outpatient diagnoses were available in Norway and Finland, and primary care diagnoses in Norway, Finland and Wales. We assessed MS case detection rate by lookback period, and compared three methods for estimating yearly MS prevalence: period prevalence (PP), average point prevalence (APP) and person-time prevalence (PTP). The estimated lookback periods to identify 95% of MS cases ranged from 6 to 9 years. APP and PTP provided lower prevalence estimates than PP, especially when the lookback to identify MS was short. In women of childbearing age, MS prevalence increased over time with all calculation methods and the highest MS prevalence was observed in Norway (PP of 402 per 100,000 in 2019). Finland showed the highest MS prevalence in pregnant women (PP of 218 per 100,000 in 2018). This study highlights the importance of sufficient lookback and available data to accurately estimate MS prevalence.

**Supplementary Information:**

The online version contains supplementary material available at https://doi.org/10.1007/s10654-025-01243-8.

## Background

Multiple sclerosis (MS) is a chronic autoimmune condition affecting the central nervous system. It is characterized by the destruction of myelin by the immune system. Women are two to four times more likely to be affected than men, with a diagnosis generally occurring during their childbearing years [1–3]. The prevalence of MS has been steadily increasing worldwide over the past few decades. Better disease management, improved diagnostic techniques, and more exhaustive case recording are the three main factors explaining this increase in prevalence [4].

In Europe, MS prevalence varies geographically, with higher prevalence observed in northern regions [5–11]. Beyond geographical differences, methodological disparities among studies also contribute to these variations. Indeed, the data sources available and the criteria used to identify the disease can significantly impact prevalence estimates. Additionally, variations in lookback windows can lead to substantial differences in prevalence estimates, highlighting the significant impact of design choices on prevalence estimation in healthcare databases [12].

In addition to the different methodologies used to identify the disease, the prevalence calculation method itself may also vary between studies, contributing to the heterogeneity of reported MS prevalences. Indeed, prevalence can be assessed at a specific point in time (point prevalence) or during a period of time (period prevalence) [12, 13]. While most epidemiological studies on MS report point prevalence, some studies calculate prevalence over a year [10]. When calculating point prevalence of a disease, the selection of the time point is crucial if the prevalence of the disease is associated with temporal changes (for example selecting a date in winter may increase estimates of the prevalence of depression). This can be overcome by averaging point prevalence measures at regular intervals during the specified period. Period prevalence can be calculated by identifying all people with the specified diagnosis during the specified period of time, which will also overcome the effect of temporal changes. However, if there is a great movement in and out of the study population, then the length of time each person is in the study needs to be included in the prevalence calculations.

Within the ConcePTION project, we aim to explore the use and safety of MS medications during pregnancy using several European healthcare data sources. The first step is to identify women with MS by comparing the prevalence estimates from different algorithms to find the most accurate one. However, calculating MS prevalence using different data sources involves several methodological choices. This study aims to explore the effect of the lookback period on MS prevalence and to compare three methods to estimate the prevalence of MS, among women of childbearing age and pregnant women, in six European healthcare data sources.

## Methods

### Study population

The study population consisted of women aged between 15 and 49 years (i.e. all women of childbearing age including pregnant women), between 2005 and 2019 from six European data sources.

### Data sources

The study was conducted using health care data sources from six European countries: Finland, Haute-Garonne (France), Emilia Romagna (Italy), Norway, Valencian Region (Spain) and Wales (UK). Detailed information on the data sources are given in online supplementary Table 1. Briefly, in Finland and Norway, data are from administrative health registries with national coverage including birth, prescription, primary and specialized health care registries. The records from all registries are linkable at the individual level by a unique national person identifier. In Haute-Garonne (France), data are from the population-based EFEMERIS cohort of pregnant women living in Haute-Garonne containing administrative health care data on pregnancy characteristics, outcomes and child health. In Emilia Romagna (Italy) and Valencian Region (Spain), data originate from regional administrative health databases and registries. They include diagnoses from hospital and specialist care contacts (only for the Italian data source) and drug dispensing data. In Wales (UK), data are linked in the SAIL databank [14, 15]; for this study, hospital admissions data (national coverage) was linked with primary care data, including all prescriptions issued in primary care. Some 85% of Wales’ primary care practices contribute data to SAIL.

The Italian, Norwegian, and Wales data sources provided data on women of childbearing age, with complete data coverage during the study period. The Spanish, Finnish, and French data sources provided data only on pregnant women. In Finland, diagnosis data from patient registries was available continuously during the study period, but prescription data was only available from three months prior to pregnancy until three months after the end of pregnancy. In Spain, diagnosis and prescription data was available continuously from 2013 to 2019. In France, the prescription data was available from 2.5 months prior to Last Menstrual Period (LMP) until the end of pregnancy and maternal diagnostic data (from inpatient data) was available only during the pregnancy.

### Study period

The study period ran from 1st January 2005 to 31st December 2019. The number of women included, the exact study periods and the median follow-up for each data source are listed in Table 1. Data may be incomplete in some databases owing to the time and nature of data collection (online supplementary Table 1). The Welsh data source included historical data from 1 January 1998 to 31 December 2004 for women resident in Wales in the study period.

**Table 1:** Study population and study period in the six data sources

Country/Region	Study population	Study period	Number of women included	Median follow-up in years (IQR^2^)
Italy/Emilia Romagna	Women of childbearing age (15–49)	01/01/2009 -31/12/2019	1,371,568	9.1 (4.5–11)
Norway/ National		01/01/2008 -31/12/2019	1,612,782	10.5 (5.5–11)
UK/Sample of Wales population (70%)		01/01/2005 -31/12/2019^1^	729,751	19.7 (14.5–22)
Finland/ National	Pregnant women aged 15–49 (data from 3 months before LMP to 3 months after end of pregnancy^3^)	01/01/2005 -31/12/2018	482,968	2.5 (1.3–4.8)
Spain/Valencian Region		01/01/2013 -31/12/2019	189,380	1.3 (1.2–1.3)
France/Haute-Garonne		01/01/2005 -31/12/2019	103,330	1 (1–3)

^1^ In Wales, additional historical data were available from 01/01/1998 for women in the study population

^2^ IQR: Interquartile range

^3^ From 2.5 months before pregnancy to end of pregnancy for the French data source

NA = Non-available

For each data source including women of childbearing age, the cohort entry date was the latest of the four following dates: the date they joined the data source, the date of their 15th birthday, 1st of Jan of the earliest year of data available in the data source or January 1st, 2005. The cohort exit date was the earliest of the four following dates: the date they left the data source, the date of death, the date of their 50th birthday or December 31st, 2019.

For the data sources including only pregnant women, we restricted data collection to 3 months before to 3 months after pregnancy to be homogeneous between these data sources: for Spain and Finland, the cohort entry date was 3 months before the 1st day of LMP of the first pregnancy and the cohort exit date was 3 months after the end of the last pregnancy; in the French data source, the cohort entry date was 2.5 months before LMP of the first pregnancy and the cohort exit date was the end of the last pregnancy. In these data sources, follow-up could contain several observation periods corresponding to the different pregnancies, separated by periods with no data available.

### Inclusion criteria

For the data sources including women of childbearing age (i.e. Italian, Norwegian, and Wales data sources), only women who had complete coverage for at least 365 consecutive days in the study period were eligible.

For the data sources only including pregnant women (i.e. Spanish, Finnish, and French data sources), all complete pregnancy periods lying within the study period for women aged between 15 and 49 years-old during the entire pregnancy period were included in the study. In the Spanish data source, the ConcePTION pregnancy algorithm was used to identify pregnancy episodes, establish the pregnancy type of end and to estimate the pregnancy start date (corresponding to the LMP date) and pregnancy end date [16].

## MS identification

Based on the results of our study comparing different algorithms to identify MS (article in print: DOI https://doi.org/10.1007/s10654-025-01264-3), MS cases were identified based on the presence of at least one MS-diagnosis or at least one dispensation (or prescription in Wales) for MS-specific Disease modifying therapies (DMT) during the study period and using the historical data from Wales. This algorithm most closely aligned with published prevalence in the countries in our study, and was used and validated in previous studies [7, 17–21]. Codes for MS diagnosis are listed in online Supplementary Table 2 and codes for MS-specific DMT are listed in online Supplementary Table 3.

## Statistical analyses according to type of data source

### Analyses conducted in data sources contributing with women of childbearing age

#### Effect of the lookback length on MS period prevalence

This analysis was conducted among women of childbearing age with more than ten years of lookback as of the 31st December 2019. The number of women with MS was assessed using lookback periods of 1, 2, 3, 5, 8 years, or the entire lookback period. The detection rate of MS cases according the lookback period was then calculated by dividing the number of MS cases identified using lookback periods of 1, 2, 3, 5, or 8 years, compared to those detected using the entire lookback period (10 years or more), in each data source. Using RStudio, logistic regressions were performed and MS cases detection rate was predicted and plotted for lookback periods ranging from 1 to 22 years, in each data source.

#### MS prevalence calculation methods

**Fig. 1:**
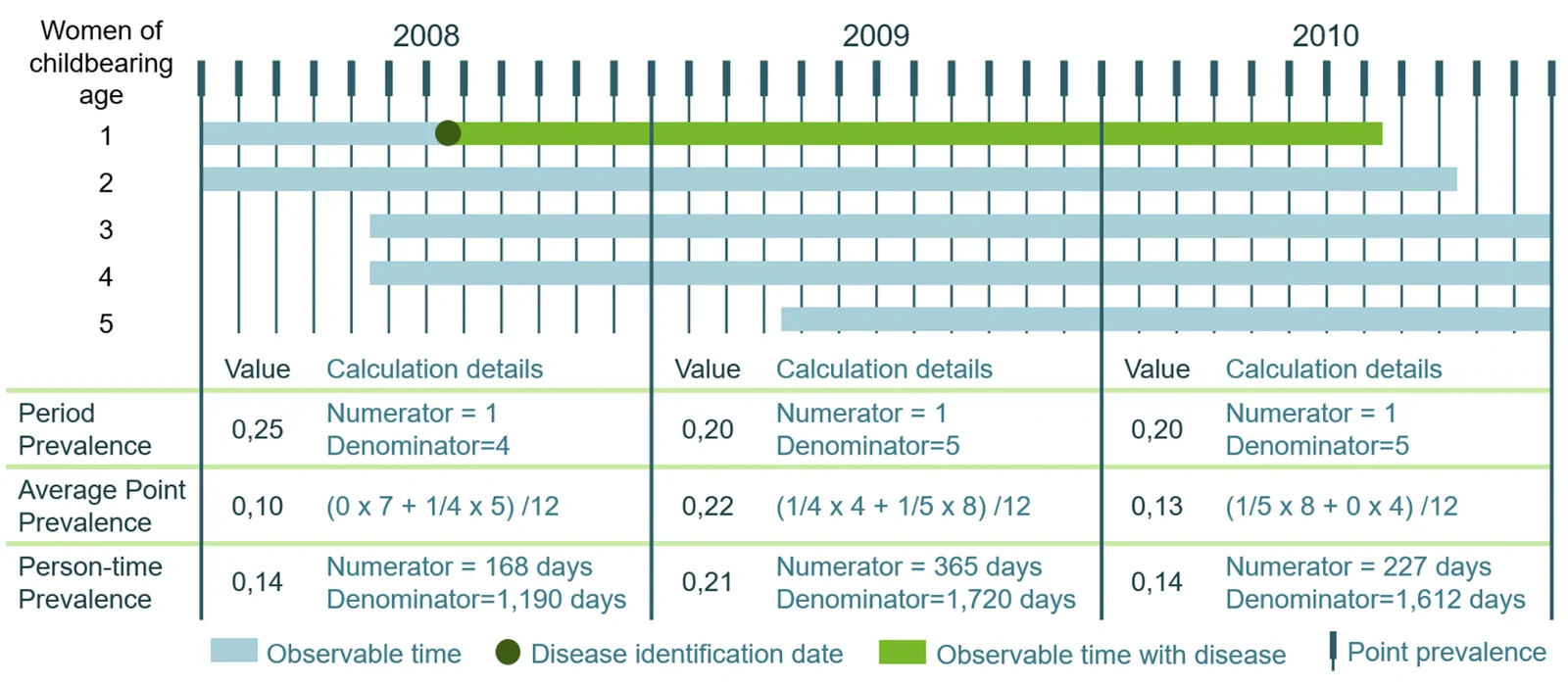
Example of prevalence calculation with three different methods among Women of childbearing age (WOCA) per year. Period prevalence is the ratio of the number of women with the disease to the number of women in the study. Average point prevalence is the average of monthly point prevalences calculated on the first day of each month. Person-time prevalence is the ratio of the number of sick days to the total number of person-days in the study

Yearly MS prevalence per 100,000 women of childbearing age was assessed over the entire study period using 3 different calculation methods: period prevalence (PP), average point prevalence (APP), person-time prevalence (PTP). Figure 1 illustrates prevalence calculation over different periods, using these 3 methods. 95% Confidence interval (95% CI) were calculated using the Wilson score method. Results were plotted by year and by calculation method, for each data source.

PP was calculated as follows: the numerator included all women observed for at least one day during each year of interest and identified with MS before the end of the year of interest and the denominator included all women observed for at least one day during the year of interest. The unit was the woman of childbearing age. Additionally, the annual PP corrected for the lack of lookback available in the early years of the data source have been estimated, using the predicted detection rate of MS cases for lookback periods ranging from 1 to 22 years. We multiplied the observed PP value by the inverse of the predicted detection rate according to the years of lookback.

APP involved calculating a point prevalence on the 1st day of each month during the year of interest; the prevalence during the year of interest was the average of all these points. The point prevalence was calculated as follows: number of women in the study and identified with MS before or on the 1st day of the month divided by the number of women in the study on the 1st day of that month. The unit of analysis was the woman in the data source population. PTP consisted in dividing the number of person-days with MS during the year of interest, by the number of person-days observable in the year of interest.

#### Analyses conducted in data sources contributing with pregnant women

The three methods to calculate MS prevalence were also computed within the data sources contributing with pregnant women, characterized by short and discontinuous follow-up periods. MS prevalence per 100,000 pregnant women was assessed by year between 2005 and 2019 using the 3 different calculation methods described above: PP, APP, PTP. 95% CI were calculated using the Wilson score method.

## Software and common data model

All Data Access Providers (DAPs) extracted an instance from their data source that was large enough to support the study design, and mapped them into the ConcePTION Common Data Model (CDM), thus obtaining an instance of the ConcePTION CDM [22]. This enabled the use of standardized analytics and tools across the network. However, the queries to be executed in distributed analyses still needed to be adapted to the diversity of the data source, including whether the data source could include all women or only pregnant women, the specific coding system, and the specific settings where diagnoses are recorded.

The script was developed using R. A script in SAS was developed to cross-check the outputs of the script within the French data source (EFEMERIS).

The DAPs executed the study code locally on their CDM instance. The result of the script was interpreted and if any inconsistencies were found the script was revised. After reviewing the aggregated results, DAPs approved their upload to the remote Research Environment hosted by the anDREa Consortium, that includes the ConcePTION partner University Medical Center Utrecht. This environment, compliant with local General Data Protection Regulation implementations, could be accessed by the principal investigator.

The results from each of the contributing data sources were then combined in tables and figures for this paper. Non-empty cell counts < 5 were shared in masked format.

## Results

### Characteristics of the study population

The number of women included in the study in each data source, and the median follow-up are provided in Table 1. The flowcharts are available in the online supplementary Fig. 1.

The number of women of childbearing age included in the study was 1,612,782 in Norway, 1,371,568 in Emilia Romagna, and 729,751 in Wales. The median follow-up (and interquartile range), including the period with historical data before entry in the study, was 19.7 years (14.5–22) in Wales, 10.5 years (5.5–12) in Norway, and 9.1 years (4.5–11) in Emilia Romagna.

The number of pregnant women included in the study was 482,968 in Finland, 103,330 in Haute-Garonne, 189,380 in the Valencian Region. The median follow-up (and interquartile range) for pregnant women was 2.5 years (1.3–4.8) in Finland, 1.0 year (1.0–3.0) in Haute-Garonne and 1.3 years (1.2–1.3) in the Valencian Region, corresponding to a median of 1 pregnancy in Haute-Garonne and Valencian Region and two pregnancies in Finland.

### Effect of the lookback length on the detection of MS cases among women of childbearing age

**Fig. 2:**
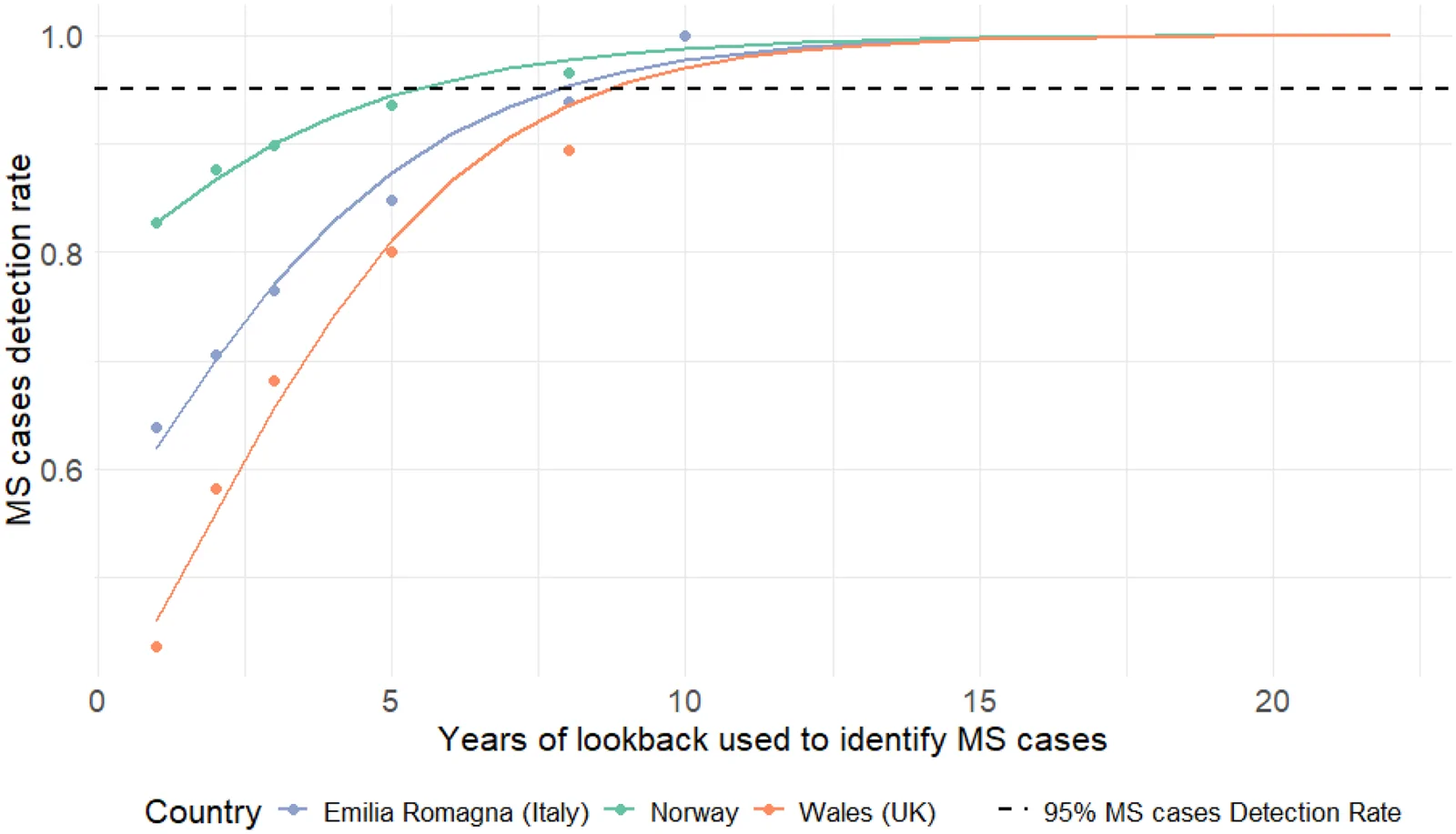
Logistic regression of MS cases detection rate according to the lookback period used to identify MS cases, in comparison with MS cases identified using the entire lookback among women of childbearing age observed from the 1st January 2010 to the 31st December 2019, in 3 healthcare data sources. Points represent observed values, and curves are the predicted values based on the logistic regression model

Among the women of childbearing age in the study between the 1st January 2010 to the 31st December 2019, 4,544, 2,477, and 934 MS cases were identified respectively in Norway, Emilia Romagna and Wales. When using only the last year of follow-up (2019) to identify MS cases, the detection rates of MS cases were respectively 83%, 64% and 44% in Norway, Emilia Romagna and Wales, in comparison with the number of cases detected using the entire lookback period (Fig. 2). The estimated lookback length required to identify 95% of MS cases was 6 years in Norway, 8 years in Emilia Romagna, and 9 years in Wales (Fig. 2). Detailed results are available in online supplementary Tables 4 and 5.

### MS prevalence by type of prevalence, and corrected prevalence by year among women of childbearing age

**Fig. 3:**
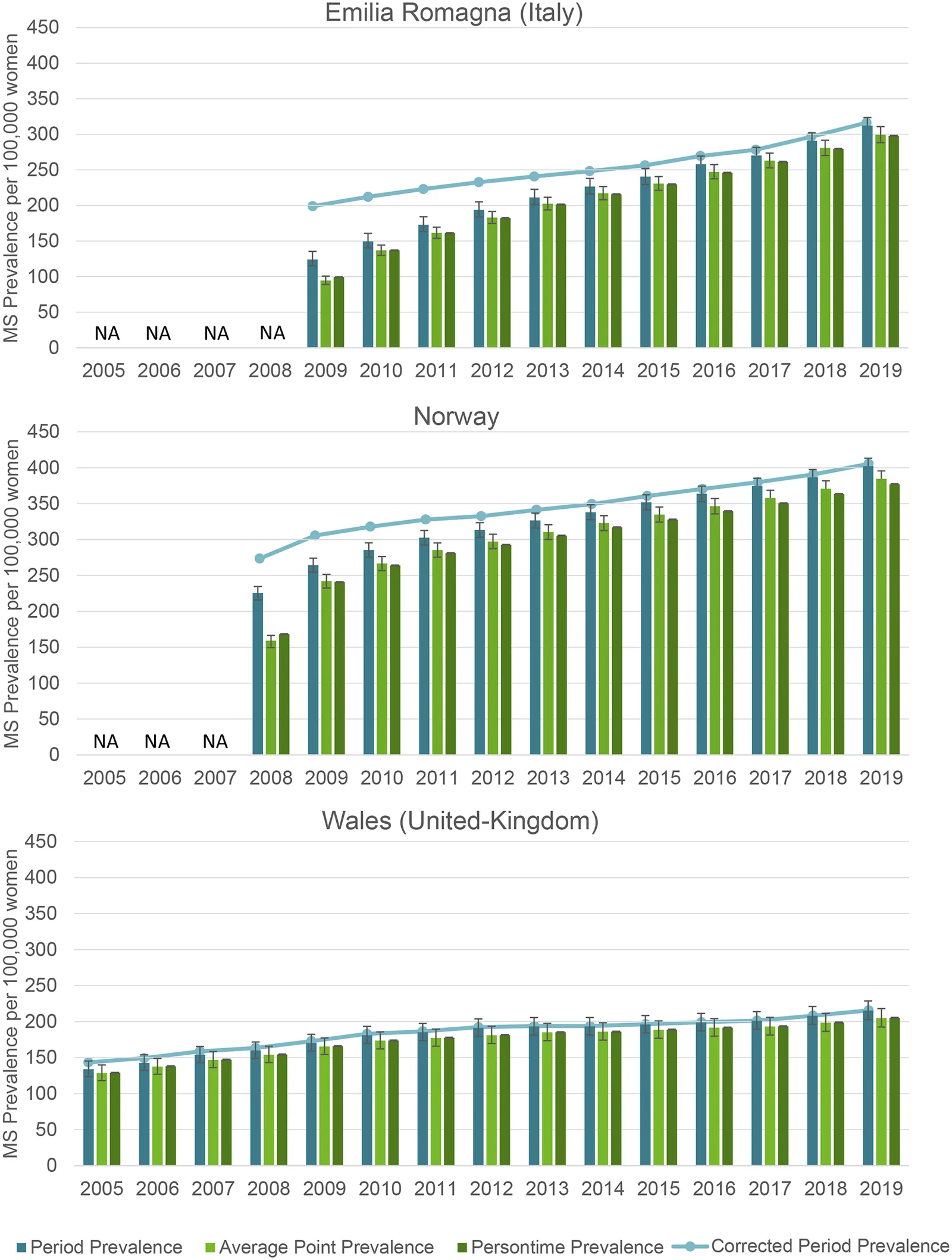
MS prevalence (95% Confidence interval) per year between 2005 and 2019 among women of childbearing age according to three types of prevalence calculation methods, and period prevalence corrected for the lack of lookback available, in three healthcare data sources. NA = Not Available. Complete results are available in online supplementary Tables 6 to 8

MS Prevalence according to the method of calculation, as well as MS PP corrected for the length of lookback available, stratified by year, within Norway, Emilia Romagna and Wales, is presented in Fig. 3. Detailed results are available in online supplementary Tables 6 to 8. In the three data sources, MS prevalence increased during the study period with all methods of calculation.

In Emilia Romagna, a total number of 3,985 women of childbearing age were identified with MS over the period 2009–2019. The highest prevalence was recorded in 2019, with a PP of 312 (95% CI: 301–324) per 100,000 women, an APP of 299 (95% CI: 288–311) per 100,000 women, and a PTP of 298 (95% CI: 297–298) per 100,000 women. In 2009, PP was 31% and 25% higher than APP and PTP respectively, and this difference decreased until 2013. From 2013 to 2019, PP was approximately 4% and 5% higher than APP and PTP respectively. PP corrected for the length of lookback available also increased from 2009 to 2019, from 200 to 318 per 100,000 women.

In Norway, a total number of 7,351 women of childbearing age were identified with MS over the period 2008–2019. In 2008, PP was 42% and 34% higher than APP and PTP respectively, and this difference decreased with time; from 2012 to 2019, PP was approximately 5% and 7% higher than APP and PTP respectively. In 2019, PP was 402 (95% CI: 391–413), APP was 385 (95% CI: 374–396) and PTP was 377 (95% CI: 377–378). PP corrected for the length of lookback available also increased from 2008 to 2019, from 273 to 405 per 100,000 women.

In Wales, a total of 1,833 women of childbearing age were identified with MS over the period 2005–2019. In 2019, PP was 216 (95% CI: 203–229), APP was 205 (95% CI: 193–218) and PTP was 205 (95% CI: 205–206). PP was approximately 4% higher than APP and PTP from 2005 to 2019. PP corrected for the length of lookback available also increased from 2005 to 2019, from 143 to 216 per 100,000 women.

### MS prevalence by type of prevalence and by year among pregnant women

**Fig. 4:**
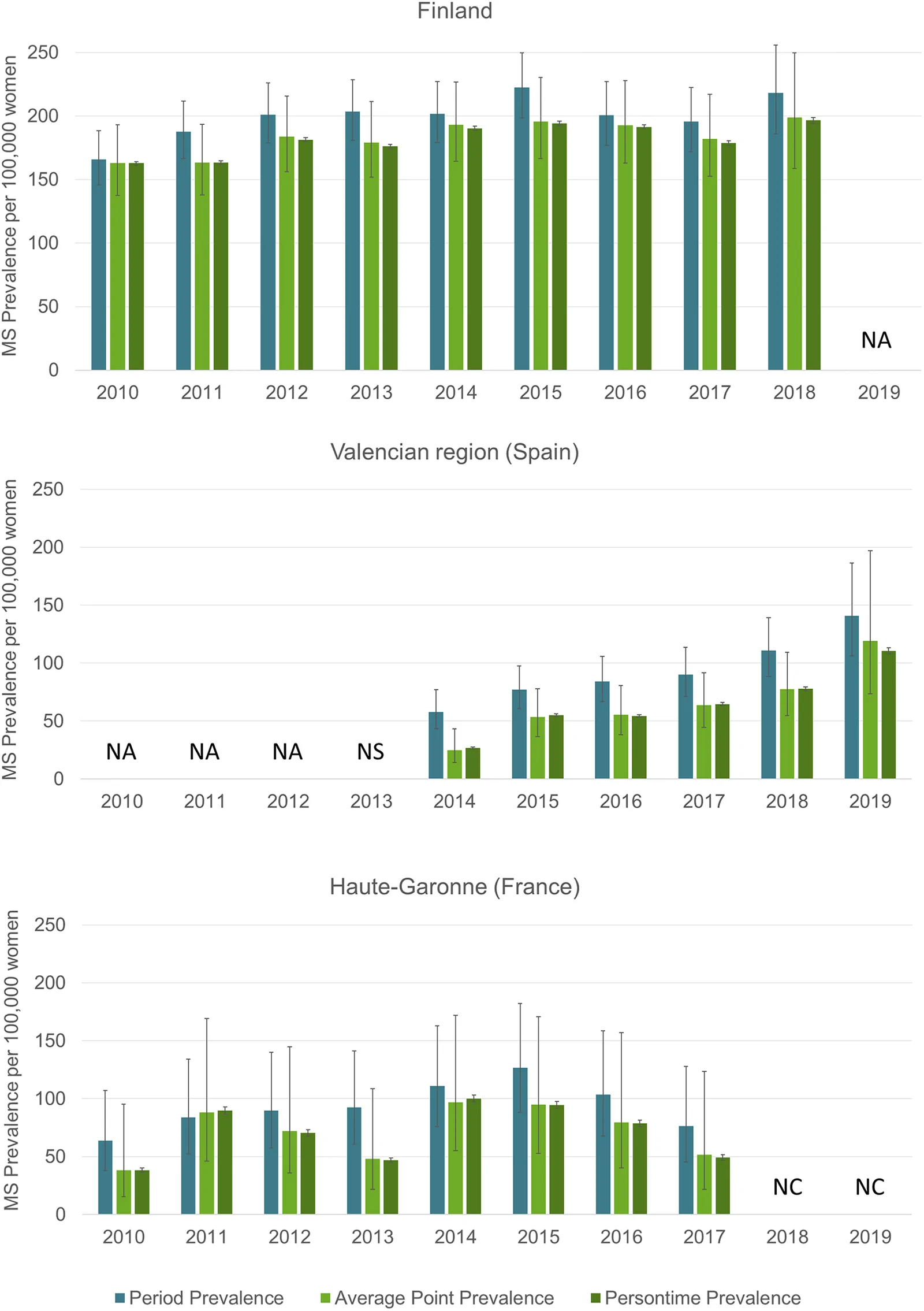
MS prevalence (95% Confidence interval) per year between 2010 and 2019 among pregnant women according to three types of prevalence calculation methods in three healthcare data sources. NA = Not Available; NC = Not Calculable. NS = Not Shown. Complete results are available in online supplementary Tables 9 to 11

MS prevalence according to the three method of calculation and stratified by year from 2010 to 2019 among pregnant women is presented in Fig. 4. Detailed results are available in online supplementary Tables 9 to 11.

In Finland, MS was identified in a total number of 1,140 pregnant women over the period 2010–2018. We observed an increase of MS prevalence from 2010 to 2015, and a slight decrease from 2015 to 2017, with the three calculation methods. In 2015, the study population included 129,394 women of whom 288 were MS cases, corresponding to a PP of 223 (95% CI: 198–250) per 100,000, an APP of 196 (95% CI: 166–230) per 100,000 pregnant women, and a PTP of 194 (95% CI: 193–196) per 100,000 pregnant women. On average over the period 2010–2018, PP was 9% higher than the APP and 11% higher than PTP, and APP was slightly higher (approximately 1%) than PTP.

In the Valencian Region, MS was identified in a total number of 220 pregnant women over the period 2013–2019. MS prevalence increased between 2014 and 2019 with the three calculation methods. The highest prevalence was recorded in 2019, with a PP of 141 (95% CI: 106–187) per 100,000 women, an APP of 119 (95% CI: 74–197) per 100,000 women, and a PTP of 111 (95% CI: 108–113) per 100,000 women. Compared to APP and PTP, PP was more than 2 times higher in 2014, approximately increased by 40% between 2015 and 2018, and by 20% in 2019. APP and PTP were similar.

In Haute-Garonne, MS was identified in a total number of 105 pregnant women over the period 2010–2019. Less than 5 cases were reported in 2018 and 2019, the prevalence was therefore not calculated in those years. The highest number of MS cases (29 cases) was observed in 2015, corresponding to a PP of 127 (95% CI: 88–182) per 100,000 women, an APP of 95 (95% CI: 53–171) per 100,000 women, and a PTP of 95(95% CI: 92–98) per 100,000 women. On average over the period 2010–2019, PP was approximately 1.4 times higher than the APP and PTP. The APP and PTP were similar.

## Discussion

### Main findings

Among women of childbearing age, the estimated lookback periods required to identify 95% of MS cases were 6 years for Norway, 8 years for Italy and 9 years for Wales. In addition, we observed an increase of MS prevalence over time, irrespective of the type of prevalence calculation method used. This increase was still observed after correcting for the lack of lookback available in the earlier years of the study. PP was significantly higher than both APP and PTP at the start of the study, but the differences progressively decreased over time.

In data sources with pregnant women, an increase in MS prevalence was observed in the Valencian Region, and until 2015 in the Finnish data source, whereas no clear increase of MS prevalence was observed in the French data source. PP was higher than both APP and PTP for the whole study period.

### Impact of lookback length on MS prevalence trends

In data sources with women of childbearing age, we observed a positive association between the length of the lookback period used to identify MS cases and MS prevalence, in line with the study of Rassen et al. [12]. When using a one-year lookback to identify MS cases compared to the entire follow-up, 83% of the cases were identified in Norway, 64% in Emilia Romagna, and 44% in Wales. These differences are probably largely due to the characteristics of the data source. Indeed, the Norwegian data source was a linkage of several national registries containing a wide range of data, including inpatient, outpatient and primary care data, explaining the high proportion of MS cases retrieved over a limited period of time. By contrast, in the Italian data source where the available data was more limited, and to a greater extent, in the Wales data source, a longer period was needed to identify the disease. In Wales, we attribute this to practitioners recording a diagnosis only once, rather than repeating entries for life-long conditions; also, hospital admission for MS is not routine, and due to funding constraints and concerns regarding serious adverse drug reactions, prescription of DMTs is particularly conservative [23, 24]. Consistently, the estimated lookback periods required to detect 95% of MS cases was 6 years for Norway, 8 years for Emilia Romagna, and 9 years for Wales.

The observed increase in MS prevalence among women of childbearing age in our data aligns with the global rise in MS prevalence in the last decades reported in the literature [4]. The rise in prevalence observed over time was still observed with the period prevalence corrected for the lack of lookback available in the early years, suggesting that this trend is not an artifact caused by lack of look back in the early years of the data.

### How to explain differences between the three types of prevalence calculation?

The PP was much higher than the APP and the PTP in the first year of the study in Emilia Romagna (2009) and Norway (2008). In these data sources, this difference decreased over time, but the PP remained higher than the other two types of prevalence until the end of the study. This important difference observed at the start of the study can be explained by the short lookback available at this stage. Indeed, for the APP calculation, the first point prevalence was calculated on the first day of the study. On this day, it was nearly impossible to identify prevalent women with MS, unless they had a prescription for MS-specific medication or a diagnosis of MS (MS identification components) reported on that particular day. The point prevalence on that day was therefore zero or very low. The next point prevalence was calculated a month after, meaning that only women having a prescription for MS-specific medication or a diagnosis of MS within this month were able to be identified as MS cases, and counted in this second point prevalence. Over the entire year, the length of time available to identify prevalent MS cases increased, making the point prevalence increasingly accurate. However, as these prevalence values were averaged over the year, the final APP over the first year reflected the initial underestimation of MS cases. Similarly, PTP, which accounts for the person-time of women identified as MS cases relative to the total person-time from women followed during the study period, was initially low due to the limited length of follow-up. Indeed, among women with MS, the person-time was accounted with MS only after MS identification. Therefore, as for the APP, the period preceding the identification of MS was considered disease-free in PTP calculation. As this disease-free period is likely due to the delay in identifying the disease following entry in the study rather than to a true disease-free period, APP and PTP would underestimate the prevalence of MS at the start of the study. By contrast, identifying the disease late in the year had no impact on the PP which accounted for the number of MS cases identified over the year relative to the number of women in the study during the year. Consistently, in Wales, where a 7-year lookback period before the start of the study was available, PP did not differ as much from APP and PTP as in Emilia Romagna and Norway.

The increasing prevalence over time is associated with an increased number of MS cases each year. These new cases can correspond to women with MS entering the study or to women who were already in the study but are newly identified with the disease. In both cases, the disease-free period prior to the disease identification in these individuals probably contribute to the constant higher PP compared to the APP and PTP, even at the end of the study.

Since MS is most often diagnosed between the ages of 20 and 40, and the women of childbearing age in our study were included from the age of 15 with long follow-ups, we can assume that a large proportion of the disease identification dates closely correspond to the timing of the MS diagnosis [25]. In these cases, considering the period before MS identification as disease-free seems more accurate. By including this disease-free period in the calculation, APP and PTP appear to be the most accurate methods. Conversely, not considering the time before MS identification as disease-free period could lead to an overestimation of MS prevalence when using PP. However, it should be acknowledged that the time lag between the actual onset of MS and its formal diagnosis, and between the diagnosis of MS and the start of DMT treatment, contribute to the imprecision of prevalence calculations, particularly when prevalence is estimated over short periods. Specifically, the process of diagnosing MS, which typically involves multiple steps such as consultations with a neurologist, brain magnetic resonance imaging exams, lumbar punctures, and blood tests often spans several months [26–28].

Interestingly, APP and PTP were closely aligned across all data sources, indicating that the precision differences between these methods had a minor impact on the results. This result was expected because women included in the study have been followed for several years, and once the disease has been identified, they remain ill until the end of their follow-up. Consequently, daily variations are very small, meaning that the day-by-day accuracy provided by PTP is already largely captured by APP.

### Pregnant women display different patterns of MS prevalence

Unlike data sources involving women of childbearing age, those with pregnant women provided data only around the pregnancy period. Consequently, pregnant women had much shorter follow-up periods, significantly limiting the time available for disease identification. During pregnancy, disease activity has been shown to decrease and some MS treatment are not recommended, also reducing the chances to detect the disease during this period [29, 30]. This might have led to an underestimation of MS cases throughout the study period, though this effect may have varied across data sources. Although the postpartum period presents a high risk of relapse or onset of the disease [31], the three months of data available after delivery in the Spanish and Finnish data sources might not be sufficient to detect the onset of a new disease. However, if these women have a subsequent pregnancy, we could detect the disease during this new pregnancy.

Moreover, the shorter follow-up period coupled with the fact that women with MS may be unlikely to start a pregnancy shortly after diagnosis reduces the chances that the disease identification date closely approximates the actual diagnosis date. Pregnant women identified with MS during the pregnancy period were therefore most likely diagnosed before the identification date, so the period preceding the date of disease identification by the algorithm is probably not a real disease-free period. We can assume that the overestimation caused by not accounting for the disease-free period with the PP method is low in this population. By contrast, considering this period as disease-free, as with the PTP and APP methods, could lead to an underestimation of MS prevalence. Therefore, in this population of pregnant women having short follow-ups, MS prevalence is probably underestimated due to the short follow-up to detect the disease, and this underestimation could be amplified with PTP and APP methods as these methods consider the period before MS identification as disease-free. However, this underestimation may vary according to the data source, depending on how observations related to the disease are recorded.

### Strengths and limitations

#### Strengths

The main strength of this study is the use of diverse data sources from multiple healthcare systems and populations with good geographic spread in Europe, improving the robustness and reliability of our findings. The consistent trends we observed between the data sources, despite the differences between their data, attest to the reliability of our approach, and supports the generalization to other data sets. The large sample sizes and long follow-ups in some of the data sources further strengthen the reliability of our findings. Also, the inclusion of all available data within each source to identify MS, particularly the comprehensive use of inpatient, outpatient, and medication records where accessible, further strengthens the accuracy of our approach.

Another strength is the comprehensive nature of our analysis, which considers different prevalence calculation methods (period prevalence, average point prevalence, and person-time prevalence). This study provided new insights into the impact of the method used to assess prevalence.

Additionally, we analysed the effect of the length of the lookback in populations with sufficient follow-up, which enabled us to understand the extent to which the length of the lookback period influences the detection of cases and further the estimation of the prevalence. This detailed analysis provides valuable insights into the temporal aspects of MS identification and prevalence estimation across Europe.

#### Limitations

A limitation of this study is the identification method for MS, which has not been manually validated using individual clinical records in the data sources used. The disease was identified based on either a diagnosis or the dispensing or prescription of MS-specific medication, which could lead to false positive cases and an overestimation of MS prevalence. However, since all three types of prevalence are similarly affected, this limitation should not impact our comparative analysis. In addition, a similar method was validated in Italian studies, and used in several studies which aimed to explore MS prevalence [7, 17–21]. Finally, our identification method, while not restrictive, had the advantage of being highly sensitive, thereby maximizing the retrieval of true MS cases.

Another limitation stems from the heterogeneity in the identifying data available across data sources. Indeed, all the data sources provided diagnoses from inpatient care, but only some of them were able to provide diagnoses from primary care or outpatient care. For example, only the Norwegian and Finnish data source provided outpatient data directly, usually referring to diagnoses made by specialists. Since patients with MS are usually followed by neurologists, this could explain, at least in part, the higher prevalence estimates in the Norwegian and Finnish data source. The lower prevalence in other data sources, especially those with pregnant women only, would then stem from a higher proportion of false negatives, and thus an underestimation of prevalence. However, the higher prevalence of MS in Finland and Norway was expected due to the known higher MS prevalence in the Nordic region [11].

## Conclusion

This study provides valuable insights into the methodological nuances involved in estimating the prevalence of a chronic disease, specifically Multiple Sclerosis (MS), among women of childbearing age and pregnant women across diverse European healthcare databases. Our results suggest that 6 to 9 years of lookback is needed to identify 95% of MS cases, depending on the amount of data available to identify the disease. Prevalence calculation methods accounting for temporal variations returned lower prevalence estimates compared to method including all identified MS cases and study participants over the period (period prevalence). Although temporally sensitive methods are more precise, they may underestimate prevalence in populations with short follow-up periods, such as pregnant women, and/or limited data. The true prevalence is likely to fall between the estimates provided by methods that account for temporal variations and those from period prevalence. It will be closer to the estimates from methods accounting for temporal variations in data sources with long follow-ups, and closer to period prevalence estimates when the follow-ups are short or data is only available during pregnancy. In 2019, within the data sources with women of childbearing age, person-days prevalence lay between 205 and 377 per 100,000 women, respectively in Wales and Norway. In 2018, within the data sources with pregnant women, period prevalence lay between 111 and 218 per 100,000, respectively in Valencian Region (Spain) and Finland. More generally, careful consideration of lookback duration and data availability is essential for accurate prevalence estimation.

## Electronic supplementary material

Below is the link to the electronic supplementary material.

Supplementary Material 1

## Data Availability

All relevant data are within the paper and its Supporting Information files. Authors may not share the study data due to regulations which restrict access and distribution to those with ethical and legal permission to use the data. The study material is available to other researchers upon an application to relevant register holders. The study protocol was registered in the HMA-EMA Catalogue (EUPAS43420) and is available on zenodo repository [32]. All code lists and scripts can be found at (https://doi.org/10.5281/zenodo.15355612).
