# Supplementary material for: Methods of estimating prevalence of multiple sclerosis in six European healthcare data sources: a contribution from the ConcePTION project
Source: Eur J Epidemiol. 2025 Jul 31;41(8):921–34. doi: 10.1007/s10654-025-01243-8 (PMC13612616; doi:10.1007/s10654-025-01243-8)
Supplement: Supplementary file 1 [file 10654_2025_1243_MOESM1_ESM.docx]

**Supplementary information**

Article title: Methods of estimating Prevalence of Multiple Sclerosis in six European healthcare data sources: A contribution from the ConcePTION project

Authors: Marie Beslay, Anna-Belle Beau, Davide Messina, Justine Benevent, Elisa Ballardini, Laia Barrachina-Bonet, Clara Cavero-Carbonell, Alex Coldea, Laura García-Villodre, Anja Geldhof, Rosa Gini, Kerstin Hellwig, Sue Jordan, Maarit K Leinonen, Sandra Lopez-Leon, Marco Manfrini, Visa Martikainen, Vera R Mitter, Amanda J Neville, Hedvig Nordeng, Aurora Puccini, Sandra Vukusic, Christine Damase-Michel, Yvonne Geissbühler, Joan K Morris

Corresponding author:

Marie Beslay, Toulouse University Hospital, CERPOP-SPHERE Team, Inserm UMR 1295, Toulouse University, Toulouse, France

beslay.m@chu-toulouse.fr

Journal: European Journal of Epidemiology

**Supplementary table 1. Description of participating data sources**

| **Country / Coverage** | **Name or registers** | **Description** |
| --- | --- | --- |
| Finland / *National* | Linkage of national registries: Medical birth registry, Prescription drug registry, National patient registry, Register of Primary Health Care visits | **Medical birth registry:** Information on births, pregnancy length, maternal parity, smoking, maternal age, maternal comorbidity, and pregnancy and birth complications.  **Prescription drug registry**: Data on all prescription entitled to reimbursement fills from pharmacies including Anatomical therapeutical chemical (ATC) classification codes and dispensing date. Over the counter medicines and medicines used during in-patient hospital stays are not included. Data are available from three months before to three months after pregnancy.  **National patient registry**: Health administrative registry from private and public specialist care. Data on inpatient stays and outpatient care contacts included diagnoses coded according to International Statistical Classification of Diseases and Related Health Problems revision 10 (ICD-10).  **Register of Primary Health Care visits:** Health administrative registry from public health care centres and increasingly from private service providers. Data on primary care visits contacts included diagnoses coded according to International Statistical Classification of Diseases and Related Health Problems revision 10 (ICD-10) or International Classification of Primary Care, second edition (ICPC-2). |
| France / *Haute-Garonne region* | EFEMERIS database | EFEMERIS database includes data on pregnant women covered by the French Health Insurance System in Haute-Garonne (south-west France). Specifically, EFEMERIS comprises data about (a) all prescriptions redeemed at pharmacies by women in ambulatory care, prior to and during pregnancy [names, Anatomical, Therapeutic and Chemical classification system (ATC) and dispensing dates]  (b) the mother (age, level of education…) and the child (gender, birth size, birth weight, congenital anomalies…) through children’s certificates filled out during the compulsory medical examinations at birth, 9 and 24 months, (c) Terminations Of Pregnancy for Fetal Anomaly (TOPFAs) that has been considered in the maternities of the region, (d) nature and date of termination of pregnancy (elective termination, stillbirth, and spontaneous abortion) from the Toulouse University Hospital centre, and (e) data on inpatient stays during pregnancy included diagnoses coded according to International Statistical Classification of Diseases and Related Health Problems revision 10 (ICD-10). |
| Italy / *Emilia Romagna region* | Linkage of Emilia-Romagna administrative databases and registries. Inhabitant registry, drug dispensations from community pharmacies and from hospital pharmacies, hospital discharge records, emergency admissions, outpatient services, exemptions from copayment, mental health services, birth registry, and death registry | Emilia-Romagna registries include birth, death, and anagraphic registries. Birth registry collect information on births, type of pregnancy, date of delivery, type of pregnancy outcome, pregnancy length, weight at birth, maternal age. Administrative databases collect information about drug dispensation from community and hospital pharmacy including ATC code and dispensing quantity and date. Hospital discharge record collect data about inpatients stay, including diagnosis (ICD9) as well as emergency admission database and mental health services databases collect data from outpatients. Moreover, exemptions from copayment databases collect information about exemption related to specific diagnostic code. |
| Norway / *National* | Linkage of national registries: Medical birth registry, Prescription drug registry, National patient registry, Register of Primary Health Care visits | **Medical birth registry:** Information on births, term data, pregnancy length, maternal parity, smoking, maternal age, maternal comorbidity, and pregnancy- and birth complications.  **Prescription drug registry (from 2005)**: Data on all prescription fills from pharmacies including Anatomical therapeutical chemical (ATC) classification codes and dispensing date. Over the counter medicines and medicines used during in-patient hospital stays are not included.  **National patient registry (from 2008)**: Health administrative registries from private and public specialist care. Data on inpatient stays in hospitals and outpatient care contacts from specialists included diagnoses coded according to International Statistical Classification of Diseases and Related Health Problems revision 10 (ICD-10).  **Register of Primary Health Care visits (from 2008):** Health administrative registries from primary care. Data on primary care contacts including outpatient specialists (data overlapping with the National patient registry). Diagnostic codes follow the International Classification of Diseases, version 10 (ICD-10) and International Classification of Primary Care (ICPC-2) which is more frequently used by general practitioners. |
| Spain / *Valencian Region* | Linkage of administrative databases and registries:  Perinatal Mortality Registry (RMPCV); Birth Registry (META-B); Congenital Anomaly Population-based Registry (RPAC-CV); Prescription and dispensations dataset (GAIA); Morbidity- Hospital discharges database (CMBD) | **Birth Registry** (for live births), **Perinatal Mortality Registry** (for stillbirths and live births deaths during the first 28 days of life) and **Congenital Anomaly Population-based Registry** (for stillbirths and live births with at least 1 major congenital anomaly): Information on births, type of pregnancy, date of delivery, type of pregnancy outcome, pregnancy length, weight at birth, maternal age.  **Prescription and dispensations** **dataset**: Data on all prescription and dispensation to outpatients entitled to reimbursement fills from pharmacies including Anatomical therapeutic chemical (ATC) classification codes and dispensing date.  **Morbidity- Hospital discharges database**: Health administrative registries from private and public inpatients care. Data on inpatient stays included diagnoses coded according to International Classification of Diseases 10th revision Spanish Version (ICD-10ES). |
| UK / *Sample of Wales population* | SAIL databank, including in-patient and primary care records, emergency care, ONS births and deaths, and data (including all prescriptions and vaccinations) from ~85% of Wales GP practices | National, whole-population data, with the exception of primary care practices. Practices contribute voluntarily, without additional funding. For details see Ford et al 2009, Lyons et al 2009. |

## ****Supplementary table 2.**** **MS codes according to coding system**

| **Coding system** | **Code** | **Text Description** |
| --- | --- | --- |
| ICD-9 | 340 | Multiple sclerosis |
| ICD-10/ ICD10ES | G35 | Multiple sclerosis |
| ICPC-2 | N86 | Multiple sclerosis |
| READ | 666A. | Multiple sclerosis review |
|  | 666B. | Multiple sclerosis multidisciplinary review |
|  | 8Cc0. | Management of multiple sclerosis in onset phase |
|  | 8Cc1. | Management of multiple sclerosis in early disease phase |
|  | 8Cc2. | Management of multiple sclerosis in stable disability phase |
|  | 8Cc4. | Management of multiple sclerosis in palliative phase |
|  | 8CS1. | Multiple sclerosis care plan agreed |
|  | 8IAb. | Multiple sclerosis review declined |
|  | 9kG. | Spec serv for pat with multiple sclerosis - enh serv admin |
|  | 9mD0. | Multiple sclerosis monitoring first letter |
|  | 9mD1. | Multiple sclerosis monitoring second letter |
|  | 9mD.. | Multiple sclerosis monitoring administration |
|  | F200. | Multiple sclerosis of the brain stem |
|  | F201. | Multiple sclerosis of the spinal cord |
|  | F202. | Generalised multiple sclerosis |
|  | F203. | Exacerbation of multiple sclerosis |
|  | F204. | Benign multiple sclerosis |
|  | F206. | Primary progressive multiple sclerosis |
|  | F207. | Relapsing and remitting multiple sclerosis |
|  | F208. | Secondary progressive multiple sclerosis |
|  | F20. | Multiple sclerosis |
|  | F20z. | Multiple sclerosis NOS |

## Supplementary table 3. Disease Modifying Therapies specific to Multiple Sclerosis

| **Medication** | **ATC Code** |
| --- | --- |
| Alemtuzumab (from 2013 onwards)^1^ | L04AA34/L04AG06 |
| Daclizumab (from 2016 onwards)^2^ | L04AC01/L04AA08 |
| Dimethyl fumarate (until end of May 2017)^3^ | N07XX09/ L04AX07 |
| Fingolimod | L04AA27/L04AE01 |
| Glatiramer acetate | L03AX13 |
| Interferon beta-1a | L03AB07 |
| Interferon beta-1b | L03AB08 |
| Ocrelizumab | L04AA36/L04AG08 |
| Peginterferon beta-1a | L03AB13 |
| Teriflunomide | L04AA31/L04AK02 |
| Siponimod | L04AA42/L04AE03 |

*^1^Between 2001 and 2012 Aletuzumab was approved by EMA to treat B-cell chronic lymphocytic leukaemia. The approval of Alemtuzumab in MS treatment by EMA was in 2013*

*^2^Between 1999 and 2009 Daclizumab was approved by EMA in organ rejection in de novo allogenic renal transplantation. The approval of Daclizumab in MS treatment by EMA was in 2016*

*^3^ In June 2017, Dimethyl Fumarate was approved to treat Psoriasis by EMA. Dimethyl Fumarate will be considered specific for MS until the end of September 2017, and non-specific from October 2017 onwards for Spain (national approval in October); for Finland (national approval 6 months before end of study period), France (not authorised) and Italy (national approval 3 months before end of study period), Dimethyl Fumarate will be considered MS-specific during the entire period.*

## Supplementary table 4. Detection rate of MS cases by lookback period in women in the study population from the 1^st^ January 2010 to the 31^st^ December 2019 in 3 healthcare data sources

|  | Norway | | | Wales (UK) | | | Italy (Emilia Romagna) | | |
| --- | --- | --- | --- | --- | --- | --- | --- | --- | --- |
| Lookback period (years) | Number of women | Number of women with MS | MS cases detection rate^1^ | Number of women | Number of women with MS | MS cases detection rate^1^ | Number of women | Number of women with MS | MS cases detection rate^1^ |
| 1 | 1,080,445 | 3,762 | 83% | 424,837 | 408 | 44% | 742,797 | 1,581 | 64% |
| 2 |  | 3,979 | 88% |  | 544 | 58% |  | 1,746 | 70% |
| 3 |  | 4,083 | 90% |  | 636 | 68% |  | 1,895 | 76% |
| 5 |  | 4,248 | 93% |  | 747 | 80% |  | 2,100 | 85% |
| 8 |  | 4,391 | 97% |  | 835 | 89% |  | 2,327 | 94% |
| ⩾ 10 |  | 4,544 | 100% |  | 934 | 100% |  | 2,477 | 100% |

^1^ MS cases detection rate = Number of women with MS / Number of women identified with MS using more than 10 years of lookback

**Supplementary figure 1.** Flowchart for the selection of women of childbearing age and pregnant women by data source

## Supplementary table 5. Predicted detection rate of MS cases for 1 to 22 years of lookback, using predictive logistic regression model based on the observed MS cases, in 3 healthcare data sources^1^

|  | | Norway | Wales (United Kingdom) | Emilia Romagna (Italy) |
| --- | --- | --- | --- | --- |
| Equation of the predictive logistic regression model | | Logit (MS detection rate) = 1.251 + 0.315 x [lookback (years)] | Logit (MS detection rate) = -0.568 + 0.405 x [lookback (years)] | Logit (MS detection rate) = 0.128 + 0.361 x [lookback (years)] |
| Lookback (years) | 1 | 83% | 46% | 62% |
|  | 2 | 87% | 56% | 70% |
|  | 3 | 90% | 66% | 77% |
|  | 4 | 92% | 74% | 83% |
|  | 5 | 94% | 81% | 87% |
|  | 6 | 96% | 87% | 91% |
|  | 7 | 97% | 91% | 93% |
|  | 8 | 98% | 94% | 95% |
|  | 9 | 98% | 96% | 97% |
|  | 10 | 99% | 97% | 98% |
|  | 11 | 99% | 98% | 98% |
|  | 12 | 99% | 99% | 99% |
|  | 13 | 100% | 99% | 99% |
|  | 14 | 100% | 99% | 99% |
|  | 15 | 100% | 100% | 100% |
|  | 16 | 100% | 100% | 100% |
|  | 17 | 100% | 100% | 100% |
|  | 18 | 100% | 100% | 100% |
|  | 19 | 100% | 100% | 100% |
|  | 20 | 100% | 100% | 100% |
|  | 21 | 100% | 100% | 100% |
|  | 22 | 100% | 100% | 100% |

**^1^** We assumed that 100% of the cases were retrieved using the entire lookback, corresponding to 10 years or more.

| Year | Number of women | Number of women with MS | Person-days | Person-days with MS | Period prevalence (95% CI) | Average point prevalence (95% CI) | Person-days prevalence (95% CI) | Maximum lookback length (years) | Predicted detection rate of MS cases | Corrected Period Prevalence |
| --- | --- | --- | --- | --- | --- | --- | --- | --- | --- | --- |
| 2009 | 997,136 | 1,237 | 355,789,811 | 353,410 | 124.1(117.3-131.2) | 94.6(89-100.7) | 99.3(99-99.7) | 1 | 62% | 200.2 |
| 2010 | 1,045,446 | 1,563 | 365,973,706 | 501,504 | 149.5(142.3-157.1) | 137(129.9-144.4) | 137(136.7-137.4) | 2 | 70% | 213.3 |
| 2011 | 1,042,458 | 1,801 | 365,194,582 | 588,659 | 172.8(165-180.9) | 161.5(153.8-169.5) | 161.2(160.8-161.6) | 3 | 77% | 224.2 |
| 2012 | 1,036,068 | 2,007 | 363,880,261 | 663,389 | 193.7(185.4-202.4) | 183.1(174.9-191.7) | 182.3(181.9-182.7) | 4 | 83% | 233.9 |
| 2013 | 1,027,172 | 2,170 | 359,322,618 | 723,675 | 211.3(202.6-220.3) | 202.5(193.8-211.6) | 201.4(200.9-201.9) | 5 | 87% | 241.8 |
| 2014 | 1,015,398 | 2,301 | 354,833,574 | 765,840 | 226.6(217.5-236) | 217.2(208.1-226.6) | 215.8(215.3-216.3) | 6 | 91% | 249.4 |
| 2015 | 1,002,301 | 2,412 | 349,978,102 | 804,070 | 240.6(231.2-250.4) | 230.8(221.4-240.6) | 229.7(229.2-230.3) | 7 | 93% | 257.5 |
| 2016 | 991,634 | 2,558 | 346,487,333 | 852,331 | 258(248.2-268.1) | 247.3(237.5-257.5) | 246(245.5-246.5) | 8 | 95% | 270.6 |
| 2017 | 980,507 | 2,649 | 341,698,895 | 893,519 | 270.2(260.1-280.6) | 263.1(252.9-273.6) | 261.5(261-262) | 9 | 97% | 279.4 |
| 2018 | 969,222 | 2,818 | 337,409,360 | 943,024 | 290.7(280.2-301.7) | 280.7(270.2-291.7) | 279.5(278.9-280.1) | 10 | 98% | 297.6 |
| 2019 | 937,708 | 2,928 | 329,121,009 | 980,225 | 312.3(301.2-323.7) | 299.3(288.3-310.8) | 297.8(297.2-298.4) | 11 | 98% | 317.5 |

## Supplementary table 6. Total number of women, number of women with MS, total person-days, person-days with MS and MS prevalence (95% Confidence interval) per year between 2005 and 2019 among women of childbearing age according to three types of prevalence calculation methods, predicted detection rate of MS cases according to the lookback, and period prevalence corrected for the lack of lookback available, in the Italian data source.

## Supplementary table 7. Total number of women, number of women with MS, total person-days, person-days with MS and MS prevalence (95% Confidence interval) per year between 2005 and 2019 among women of childbearing age according to three types of prevalence calculation methods, predicted detection rate of MS cases according to the lookback, and period prevalence corrected for the lack of lookback available, in the Norwegian data source.

| Year | Number of women | Number of women with MS | Person-days | Person-days with MS | Period prevalence (95% CI) | Average point prevalence (95% CI) | Person-days prevalence (95% CI) | Maximum lookback length (years) | Predicted detection rate of MS cases | Corrected Period Prevalence |
| --- | --- | --- | --- | --- | --- | --- | --- | --- | --- | --- |
| 2008 | 1,093,408 | 2,467 | 389,587,928 | 655,591 | 225.6(216.9-234.7) | 159.1(152.1-166.5) | 168.3(167.9-168.7) | 1 | 83% | 272.9 |
| 2009 | 1,146,206 | 3,032 | 403,196,677 | 970,721 | 264.5(255.3-274.1) | 242.2(233.2-251.5) | 240.8(240.3-241.2) | 2 | 87% | 304.9 |
| 2010 | 1,165,293 | 3,327 | 410,670,137 | 1,083,975 | 285.5(276-295.4) | 266.7(257.4-276.4) | 264(263.5-264.4) | 3 | 90% | 317.3 |
| 2011 | 1,184,691 | 3,585 | 417,511,873 | 1,173,796 | 302.6(292.9-312.7) | 285.4(275.8-295.3) | 281.1(280.6-281.6) | 4 | 93% | 327.2 |
| 2012 | 1,202,600 | 3,768 | 425,209,624 | 1,244,315 | 313.3(303.5-323.5) | 297.3(287.6-307.4) | 292.6(292.1-293.1) | 5 | 94% | 331.9 |
| 2013 | 1,219,520 | 3,983 | 430,102,332 | 1,313,672 | 326.6(316.6-336.9) | 310.5(300.6-320.7) | 305.4(304.9-306) | 6 | 96% | 340.7 |
| 2014 | 1,234,962 | 4,175 | 435,577,809 | 1,381,229 | 338.1(328-348.5) | 322.9(312.9-333.2) | 317.1(316.6-317.6) | 7 | 97% | 348.8 |
| 2015 | 1,248,714 | 4,394 | 440,511,321 | 1,444,100 | 351.9(341.6-362.4) | 334.8(324.7-345.3) | 327.8(327.3-328.4) | 8 | 98% | 360.0 |
| 2016 | 1,260,940 | 4,587 | 446,326,754 | 1,515,413 | 363.8(353.4-374.4) | 346.5(336.2-357.1) | 339.5(339-340.1) | 9 | 98% | 369.9 |
| 2017 | 1,270,134 | 4,758 | 448,698,864 | 1,572,609 | 374.6(364.1-385.4) | 357.8(347.4-368.6) | 350.5(349.9-351) | 10 | 99% | 379.2 |
| 2018 | 1,278,067 | 4,942 | 451,515,209 | 1,641,326 | 386.7(376.1-397.6) | 370.9(360.4-381.8) | 363.5(363-364.1) | 11 | 99% | 390.2 |
| 2019 | 1,271,576 | 5,114 | 451,527,554 | 1,702,665 | 402.2(391.3-413.3) | 384.6(373.8-395.6) | 377.1(376.5-377.7) | 12 | 99% | 404.8 |

## Supplementary table 8. Total number of women, number of women with MS, total person-days, person-days with MS and MS prevalence (95% Confidence interval) per year between 2005 and 2019 among women of childbearing age according to three types of prevalence calculation methods, predicted detection rate of MS cases according to the lookback, and period prevalence corrected for the lack of lookback available, in the Welsh data source.

| Year | Number of women | Number of women with MS | Person-days | Person-days with MS | Period prevalence (95% CI) | Average point prevalence (95% CI) | Person-days prevalence (95% CI) | Maximum lookback length (years) | Predicted detection rate of MS cases | Corrected Period Prevalence |
| --- | --- | --- | --- | --- | --- | --- | --- | --- | --- | --- |
| 2005 | 437,241 | 586 | 153,977,950 | 198,501 | 134(123.6-145.3) | 128.5(118.2-139.8) | 128.9(128.3-129.5) | 8 | 94% | 143.2 |
| 2006 | 453,598 | 647 | 159,946,921 | 220,757 | 142.6(132.1-154.1) | 137.5(127-149) | 138(137.4-138.6) | 9 | 96% | 149.1 |
| 2007 | 468,109 | 720 | 165,136,842 | 243,072 | 153.8(143-165.5) | 146.9(136.1-158.4) | 147.2(146.6-147.8) | 10 | 97% | 158.5 |
| 2008 | 479,958 | 768 | 169,907,213 | 262,297 | 160(149.1-171.7) | 154(143.2-165.7) | 154.4(153.8-155) | 11 | 98% | 163.3 |
| 2009 | 489,521 | 834 | 172,860,016 | 286,758 | 170.4(159.2-182.3) | 165.4(154.2-177.4) | 165.9(165.3-166.5) | 12 | 99% | 172.7 |
| 2010 | 496,674 | 900 | 175,223,461 | 304,500 | 181.2(169.8-193.4) | 173.6(162.2-185.7) | 173.8(173.2-174.4) | 13 | 99% | 182.8 |
| 2011 | 503,106 | 932 | 176,894,372 | 314,217 | 185.2(173.7-197.5) | 177.3(165.9-189.6) | 177.6(177-178.3) | 14 | 99% | 186.3 |
| 2012 | 509,487 | 975 | 179,175,110 | 324,845 | 191.4(179.7-203.8) | 181.1(169.6-193.4) | 181.3(180.7-181.9) | 15 | 100% | 192.2 |
| 2013 | 513,415 | 992 | 179,921,111 | 333,261 | 193.2(181.6-205.6) | 185.2(173.6-197.6) | 185.2(184.6-185.9) | 16 | 100% | 193.7 |
| 2014 | 516,149 | 998 | 180,686,447 | 336,046 | 193.4(181.7-205.7) | 186(174.3-198.4) | 186(185.4-186.6) | 17 | 100% | 193.7 |
| 2015 | 516,766 | 1,013 | 180,950,259 | 341,686 | 196(184.3-208.5) | 188.6(176.9-201.1) | 188.8(188.2-189.5) | 18 | 100% | 196.2 |
| 2016 | 516,960 | 1,028 | 181,257,927 | 347,385 | 198.9(187.1-211.4) | 191.6(179.8-204.2) | 191.7(191-192.3) | 19 | 100% | 199.1 |
| 2017 | 515,341 | 1,037 | 180,045,994 | 348,447 | 201.2(189.4-213.8) | 193.3(181.4-205.9) | 193.5(192.9-194.2) | 20 | 100% | 201.3 |
| 2018 | 512,618 | 1,067 | 177,983,363 | 353,732 | 208.1(196-221) | 198.6(186.5-211.4) | 198.7(198.1-199.4) | 21 | 100% | 208.1 |
| 2019 | 497,505 | 1,072 | 174,440,392 | 358,097 | 215.5(203-228.8) | 204.9(192.5-218.1) | 205.3(204.6-206) | 22 | 100% | 215.5 |

## Supplementary table 9. Total number of women, number of women with MS, total person-days, person-days with MS and MS prevalence (95% Confidence interval) per year between 2010 and 2019 among women of childbearing age according to three types of prevalence calculation methods, in the Finish data source.

| Year | Number of women | Number of women with MS | Person-days | Person-days with MS | Period prevalence (95% CI) | Average point prevalence (95% CI) | Person-days prevalence (95% CI) |
| --- | --- | --- | --- | --- | --- | --- | --- |
| 2005 | 66,110 | 74 | 12,106,538 | 9,515 | 111.9(89.2-140.5) | 61.2(40.6-267.7) | 78.6(77-80.2) |
| 2006 | 125,016 | 171 | 27,947,339 | 32,107 | 136.8(117.8-158.9) | 116.3(94.4-143.2) | 114.9(113.6-116.1) |
| 2007 | 138,108 | 204 | 29,123,979 | 36,376 | 147.7(128.8-169.4) | 126.3(103.9-153.4) | 124.9(123.6-126.2) |
| 2008 | 140,165 | 210 | 29,586,212 | 39,746 | 149.8(130.9-171.5) | 135.6(112.5-163.5) | 134.3(133-135.7) |
| 2009 | 141,592 | 231 | 29,909,205 | 42,114 | 163.1(143.4-185.6) | 142.3(118.7-170.6) | 140.8(139.5-142.2) |
| 2010 | 141,739 | 235 | 29,896,638 | 48,662 | 165.8(145.9-188.4) | 162.8(137.5-192.9) | 162.8(161.3-164.2) |
| 2011 | 141,185 | 265 | 29,707,525 | 48,515 | 187.7(166.4-211.7) | 163.4(137.9-193.6) | 163.3(161.9-164.8) |
| 2012 | 138,848 | 279 | 29,311,440 | 53,148 | 200.9(178.7-225.9) | 183.6(156.3-215.7) | 181.3(179.8-182.9) |
| 2013 | 136,693 | 278 | 28,719,222 | 50,592 | 203.4(180.9-228.7) | 179.2(152-211.3) | 176.2(174.6-177.7) |
| 2014 | 133,866 | 270 | 28,051,002 | 53,370 | 201.7(179-227.2) | 193.2(164.5-226.7) | 190.3(188.7-191.9) |
| 2015 | 129,394 | 288 | 27,049,017 | 52,521 | 222.6(198.3-249.8) | 195.7(166.4-230.2) | 194.2(192.5-195.8) |
| 2016 | 124,176 | 249 | 25,859,279 | 49,495 | 200.5(177.1-227) | 192.7(163-227.8) | 191.4(189.7-193.1) |
| 2017 | 117,530 | 230 | 24,658,484 | 44,091 | 195.7(172-222.6) | 181.9(152.5-217) | 178.8(177.1-180.5) |
| 2018 | 68,773 | 150 | 14,551,042 | 28,590 | 218.1(185.9-255.9) | 198.8(158.5-249.6) | 196.5(194.2-198.8) |

## Supplementary table 10. Total number of women, number of women with MS, total person-days, person-days with MS and MS prevalence (95% Confidence interval) per year between 2010 and 2019 among women of childbearing age according to three types of prevalence calculation methods, in the French data source. NC= Not Calculable.

| Year | Number of women | Number of women with MS | Person-days | Person-days with MS | Period prevalence (95% CI) | Average point prevalence (95% CI) | Person-days prevalence (95% CI) |
| --- | --- | --- | --- | --- | --- | --- | --- |
| 2005 | 10,215 | <5 | 1,834,713 | 38 | NC | NC | 2.1(1.5-2.8) |
| 2006 | 20,115 | 8 | 3,601,114 | 1,052 | 39.8(20.2-78.5) | 27.9(9.3-85.1) | 29.2(27.5-31) |
| 2007 | 16,328 | 8 | 3,393,178 | 631 | 49(24.8-96.7) | 20.7(5.4-76.7) | 18.6(17.2-20.1) |
| 2008 | 8,077 | <5 | 6,27,279 | 87 | NC | NC | 13.9(11.2-17.1) |
| 2009 | 14,592 | 7 | 2,941,204 | 1521 | 48(23.2-99) | 49.8(19.4-142.7) | 51.7(49.2-54.4) |
| 2010 | 21,969 | 14 | 4,047,382 | 1,556 | 63.7(38-106.9) | 38.2(15.4-95.4) | 38.4(36.6-40.4) |
| 2011 | 20,287 | 17 | 3,569,689 | 3,199 | 83.8(52.3-134.2) | 88.1(46.2-169.3) | 89.6(86.6-92.8) |
| 2012 | 21,199 | 19 | 3,834,297 | 2,700 | 89.6(57.4-140) | 71.9(35.8-144.7) | 70.4(67.8-73.1) |
| 2013 | 22,694 | 21 | 4,063,742 | 1,903 | 92.5(60.5-141.4) | 47.9(21.7-108.6) | 46.8(44.8-49) |
| 2014 | 23,394 | 26 | 4,265,185 | 4,267 | 111.1(75.9-162.8) | 97(55-171.8) | 100(97.1-103.1) |
| 2015 | 22,863 | 29 | 4,138,819 | 3,910 | 126.8(88.3-182.1) | 95(53-170.8) | 94.5(91.6-97.5) |
| 2016 | 20,260 | 21 | 3,649,052 | 2,876 | 103.7(67.8-158.4) | 79.5(40.4-156.9) | 78.8(76-81.7) |
| 2017 | 18,358 | 14 | 3,316,709 | 1,635 | 76.3(45.4-128) | 51.6(21.9-123.5) | 49.3(47-51.7) |
| 2018 | 19,201 | <5 | 3,485,130 | 160 | NC | NC | 4.6(3.9-5.4) |
| 2019 | 10,239 | <5 | 1,824,882 | 682 | NC | NC | 37.4(34.7-40.3) |

## Supplementary table 11. Total number of women, number of women with MS, total person-days, person-days with MS and MS prevalence (95% Confidence interval) per year between 2013 and 2019 among women of childbearing age according to three types of prevalence calculation methods, in the Spanish data source.

| Year | Number of women | Number of women with MS | Person-days | Person-days with MS | Period prevalence (95% CI) | Average point prevalence (95% CI) | Person-days prevalence (95% CI) |
| --- | --- | --- | --- | --- | --- | --- | --- |
| 2013 | 40,261 | 6 | 7,300,669 | 1,153 | 14.9(6.8-32.5) | 14.8(5.2-331.9) | 15.8(14.9-16.7) |
| 2014 | 79,691 | 46 | 17,860,538 | 4,795 | 57.7(43.3-77) | 24.8(14.3-43.2) | 26.8(26.1-27.6) |
| 2015 | 88,375 | 68 | 18,130,378 | 9,969 | 76.9(60.7-97.5) | 53.5(36.7-78) | 55(53.9-56.1) |
| 2016 | 85,678 | 72 | 17,637,870 | 9,608 | 84(66.7-105.8) | 55.3(38-80.6) | 54.5(53.4-55.6) |
| 2017 | 78,861 | 71 | 16,339,006 | 10,564 | 90(71.4-113.5) | 63.8(44.5-91.7) | 64.7(63.4-65.9) |
| 2018 | 67,570 | 75 | 14,835,108 | 11,549 | 111(88.6-139.1) | 77.5(54.8-109.5) | 77.8(76.4-79.3) |
| 2019 | 34,099 | 48 | 6,325,480 | 7,003 | 140.8(106.2-186.6) | 119(73.7-196.9) | 110.7(108.1-113.3) |
